# Ultrasound and shock-wave stimulation to promote axonal regeneration following nerve surgery: a systematic review and meta-analysis of preclinical studies

**DOI:** 10.1038/s41598-018-21540-5

**Published:** 2018-02-16

**Authors:** Simeon C. Daeschler, Leila Harhaus, Philipp Schoenle, Arne Boecker, Ulrich Kneser, Konstantin D. Bergmeister

**Affiliations:** Department of Hand, Plastic and Reconstructive Surgery, Burn Center, Department of Plastic and Hand Surgery, University of Heidelberg, BG Trauma Hospital Ludwigshafen, Ludwigshafen, Germany

## Abstract

Limited regeneration after nerve injury often leads to delayed or incomplete reinnervation and consequently insufficient muscle function. Following nerve surgery, application of low-intensity ultrasound or extracorporeal shock waves may promote nerve regeneration and improve functional outcomes. Because currently clinical data is unavailable, we performed a meta-analysis following the PRISMA-guidelines to investigate the therapeutic effect of ultrasound and shock wave therapies on motor nerve regeneration. Ten ultrasound-studies (N = 445 rats) and three shock-wave studies (N = 110 rats) were identified from multiple databases. We calculated the difference in means or standardized mean difference with 95% confidence intervals for motor function, nerve conduction velocity and histomorphological parameters of treated versus sham or non-treated animals. Ultrasound treatment showed significantly faster nerve conduction, increased axonal regeneration with thicker myelin and improved motor function on sciatic functional index scale (week two: DM[95%CI]: 19,03[13,2 to 25,6], 71 animals; week four: 7,4[5,4 to 9,5], 47 animals). Shock wave induced recovery improvements were temporarily significant. In conclusion, there is significant evidence for low-intensity ultrasound but not for extracorporeal shock wave treatment to improve nerve regeneration. Prospective clinical trials should therefore investigate available FDA-approved ultrasound devices as adjunct postoperative treatment following nerve surgery.

## Introduction

The surgical treatment of traumatic peripheral nerve injuries is complex and still leads to impaired function in a significant number of patients^1–4^. Despite improved understanding of the pathophysiology, the key issue of slow nerve regeneration remains unsolved and often leads to a delayed and incomplete reinnervation with consecutive muscle fibrosis. This results in considerable functional disability in mostly young and previously healthy adult patients^2,3^. From the socioeconomic perspective, nerve injuries are associated with long periods of recovery, sick leave and sometimes life-long functional disability, which result in a high economic burden for both patient and society^2,5^. Peripheral nerve injuries are usually reconstructed by either primary repair or, where tension-free coaptation is impossible, using artificial conduits or the gold standard autologous nerve grafts. Consequently, the frequently long regeneration distances between lesion and end organ represent a limiting factor for sufficient reinnervation^6–8^. One of the approaches to accelerate peripheral nerve regeneration is to stimulate the physiological processes that occur following nerve injury. Physiologically, severe axonal damage leads to Wallerian degeneration of the nerve distal to the lesion and, if surgically reconnected, ideally to subsequent axonal regeneration and remyelination. Schwann cells play a pivotal role in these processes since they are involved in macrophage recruitment and phagocytosis of the cellular debris following axonal breakdown^9^. Moreover, during the proliferative phase, they provide structural guidance and trophic support to the regenerating axons^10,11^. Repetitive external stimulation via low-intensity ultrasound therapy (US) or extracorporeal shock wave treatment (ESWT) was postulated to enhance these cellular repair mechanisms after nerve surgery. Ultrasound waves induce mechanical motion of molecules in periodically alternating phases of compression and rarefaction and have thereby shown to stimulate tissue regeneration due to transmission of mechanical energy^12–14^. US is clinically well established in the treatment of bone fractures^12^ and has been shown to significantly promote regeneration of ligaments and articular cartilage in intervertebral discs^13–16^. In contrast, extracorporeal shock waves are single, predominantly positive pressure waves with high amplitudes, short duration and rapid rise times. For lower energies, there is evidence to improve regeneration in several tissues such as wounds, ulcers, burned skin, ischemic myocardium and others^17–20^. Moreover, ESWT is clinically used to treat nonunion fractures^21^. Currently, no clinical studies exist examining the effects of US and ESWT on nerve regeneration. However, several experimental studies have investigated their use as an adjunct treatment following peripheral motor nerve repair. This study presents the first systematic review of the available preclinical literature as well as meta-analyses of the reported effects and their potential clinical application.

## Results

### Results of the search

**Figure 1:**
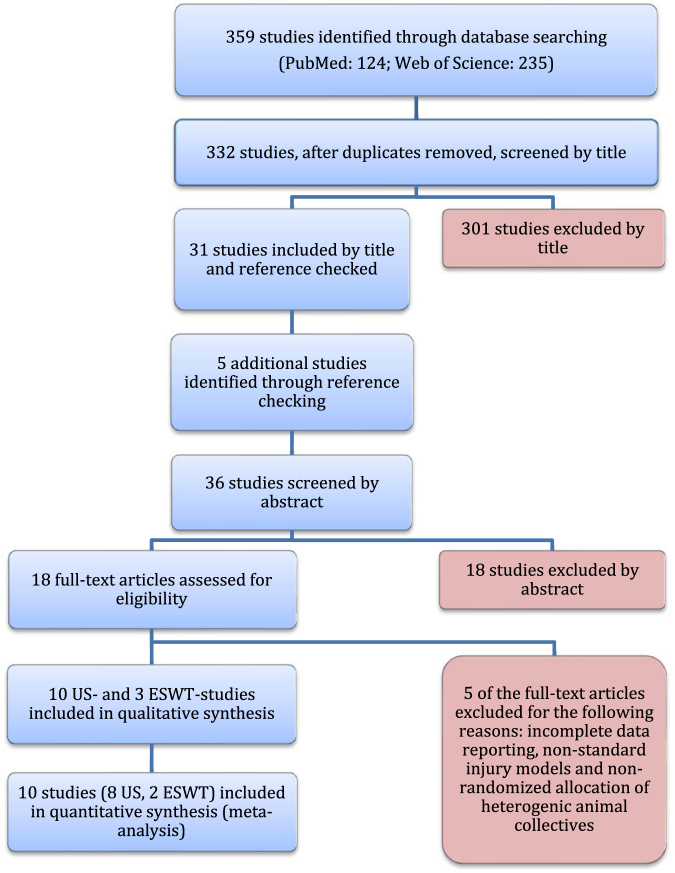
Flow diagram. Visualization of the literature search and the study selection process according to the PRISMA guidelines.

A systematic literature search according to the PRISMA-guidelines was performed on October 3^rd^, 2016 and identified a total of 359 studies, preclinically investigating either US or EWST in the context of peripheral nerve regeneration. After discarding of duplicates, 332 studies remained of which 31 were eligible for inclusion after title screening. Thereby, five additional eligible studies were identified from the studies’ references. In summary, following subsequent abstract and full-text screening, ten ultrasound studies and three extracorporeal shock wave studies fulfilled the inclusion criteria (Table 1). Figure 1 illustrates the study selection process.

**Table 1:** Inclusion criteria for studies for this review.

	Inclusion criteria
Population	• Peripheral motor nerve lesions in rat
Intervention	• Extracorporeal shock wave treatment (ESWT)• Low-intensity ultrasound therapy (US)
Comparison	• Sham• No intervention
Outcome	• Voluntary motor function• Nerve conduction velocity (NCV), compound muscle action potential (CMAP)• Distal nerve fiber count or density, myelin sheath thickness, nerve fiber diameter or axon diameter
Study design	• Experimental animal study• English or German language

This table presents the inclusion criteria for this systematic review based on the PICOS aspects (participants, interventions, comparators, outcomes, and study design) as recommended by the Cochrane Handbook for Systematic Reviews.

### Study characteristics

All 13 included preclinical studies were published in English and used the standard rat sciatic nerve model in a total of 555 animals (US: N = 445 rats; ESWT: N = 110). The sample size varied from 20^22,23^ to 60^24,25^ animals. Six^23,25–27^ of the 13 studies used a nerve crush (axotomy) as injury model, resulting in disruption of the axons continuity but preservation of the nerves peri- and epineurium^28^. Two^24,29^ of the 13 studies used reverse sciatic autografts (8 mm and 10 mm) with epineural sutures and five other studies^22,30–33^ reconstructed a 10–15 mm sciatic nerve gap via nerve conduits of 12–17 mm length and 1,0–1,6 mm inner diameter. The implanted nerve conduits were tubes of biodegradable, microporous polylactic acid^22,30,31,33^ or polycaprolactone^32^ membranes or non-biodegradable silicone conduits in two groups^30,31^.

US was applied during the first 24–72 h post-injury and continued in varying intervals from daily^22–25^ to weekly^32,33^ and for one to five minutes respectively. US-intensities in included treatment groups ranged from 200 to 500 mW/cm^2^ and frequencies varied from 1,0 to 3,3 MHz with a majority using 1,0–1,1MHz^22–27,30–33^. In consequence of this heterogeneity across the included US-studies and since previous reports detected significant dose-dependent differences in outcomes of interest, we clustered the US-studies in two groups according to their application intensities, ranging from 200–300 mW/cm^2^ and 400–500 mW/cm^2^ to enable dose-dependent comparison and to potentially identify the most effective intensities^24^.

ESWT was applied either once^29^ immediately after injury or six^34^ or ten^35^ times during 14 days post-injury in the following doses: 300 impulses with 3 Hz frequency and 0,09–0,1 mJ/mm^2^ energy. Evaluation periods ranged in US-studies from three^23,33^ to 24 weeks^32^ and in ESWT-studies from two^34^ to 12 weeks^29^. Outcomes of interest included voluntary motor function on sciatic functional index scale (SFI-scale), neuro-histomorphological parameters (diameter of myelinated axons, myelin thickness as well as density or number of myelinated nerve fibers distal to the lesion site or inside the implanted interponate) and neurophysiological parameters such as nerve conduction velocity (NCV) or compound muscle action potential (CMAP) of target muscles. Table two provides an overview of the key elements of all included studies.

### Effects of interventions

#### Ultrasound intensity of 200–300 mW/cm^2^

Seven studies investigated repetitive US with an intensity of 200–300 mW/cm^2^ and frequencies between 1 MHz and 3,3 MHz. The application was conducted either continuously for one or two minutes, or using pulsed output (20% duty cycle) for five minutes. Control groups consisted of sham or no intervention groups. Five studies reported motor function in a total of 92 rats. Voluntary motor function of ultrasound treated animals was significantly improved starting two weeks following axonotmetic injury^25–27^ and four weeks following reverse autograft or nerve conduit implantation^22,24^. These improvements remained significant for the entire two or three months evaluation period^22,24,25^.

Four studies including a total of 170 rats reported histomorphometric parameters including the density, number, diameter and myelin sheath thickness of myelinated nerve fibers. Following crush injury, ultrasound treated animals had significantly more axons per area 4 mm distal to the lesion site compared to control animals (p < 0,01)^25^. Animals treated with autograft repair following 10 mm nerve defects showed significantly higher values (p < 0,01) in fiber density, total nerve fiber diameter and myelin thickness of myelinated axons in the mid-sections of the autografts at twelve weeks post-surgery, if repeatedly treated with US^24^. In reconstructions using implantation of polylactic acid conduits, US treatment resulted in significantly more myelinated axons with significantly larger individual cross-sectional area inside the conduit^30,31^. Four of seven studies, including a total of 124 rats, reported neurophysiological analyses. Here, axotomized rats receiving US had significantly faster (p < 0,01) nerve conduction velocities starting from week four on^25^ and significantly higher CMAP^24,26^. Likewise, animals with custom made polylactic acid nerve conduits showed significantly faster nerve conduction velocities following repetitive ultrasound sonication^22^. Neurophysiological analyses were not conducted in any of the autograft-repair groups.

#### Ultrasound intensity of 400–500 mW/cm^2^

Five studies investigated repetitive ultrasound treatment with an intensity of 400–500 mW/cm^2^ and a frequency of 1 MHz. The application was conducted in pulsed mode (20% duty cycle) for two to five minutes and sham or no intervention groups served as a control. Two of these studies with a total of 30 animals analyzed motor function, reporting that animals exposed to US had significantly improved voluntary motor function (p ≤ 0,02) beginning at two weeks after crush injury^23^ and four weeks respectively after sciatic autograft-repair^24^.

Histomorphological effects of US treatment were investigated in four studies including a total of 78 animals. Here, repeated application of US resulted in significantly higher counts of myelinated axons after nerve crush^23^ and significantly higher (p ≤ 0,01) density, diameter and myelin sheath thickness of myelinated nerve fibers twelve weeks after autograft repair^24^. Likewise, US resulted in significantly thicker nerve fibers and myelin sheaths four weeks after repair using biodegradable nerve conduits^33^. These improvements remained significant six months after surgery^32^.

Two studies, investigating US in 22 animals, reported that neurophysiological results (CMAP-area under the curve of operated side divided by those of the non-operated contralateral side) in autografted animals were significantly (p < 0,01) improved compared to control^24^. Likewise, in rats which received US following nerve reconstruction via biodegradable conduits, nerve conduction was significantly improved up to six months following surgery^32^.

#### Extracorporeal shock wave therapy (ESWT)

Three studies compared repetitive and single ESWT using 300 impulses with a frequency of 3 Hz and the energy of 0,09–0,1 mJ/mm^2^ with sham or non-treated controls. All included shock wave studies totaling 110 animals reported voluntary motor function as an outcome parameter. Following repetitive ESWT, rats with sciatic nerve crush had significantly improved voluntary motor function as early as two weeks after surgery compared to control^34,35^. If ESWT was applied only once following sciatic nerve reconstruction via reversed autograft, voluntary motor function was significantly improved compared to control at weeks four to ten, with no significant difference remaining at 12 weeks post-injury^29^.

The same study compared the histomorphology in 20 single ESW-treated animals with control and reported significantly increased myelinated nerve fiber numbers at three weeks after surgery, which again was not evident at 12 weeks^29^. In contrast, nerve conduction velocity in 40 animals of this study was significantly increased three months after surgery^29^.

### Meta-analysis

**Figure 2:**
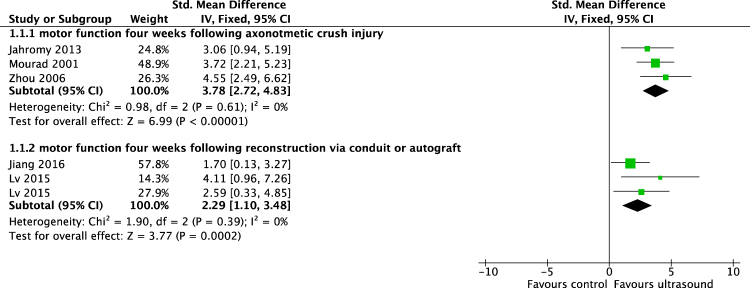
Meta-analysis. To compare the different scales used to measure identical outcome parameters in the included studies, we calculated the standardized mean difference (SMD) and calculated the DM afterwards in accordance with the Cochrane Handbook for Systematic Reviews. Shown are the standardized mean differences of walking track performance of animals treated with an ultrasound intensity of 200–300 mW/cm^2^ compared to sham or untreated animals four weeks following axonotmetic nerve injury and four weeks following implantation of biodegradable synthetic nerve conduit or reverse autograft.

**Figure 3:**
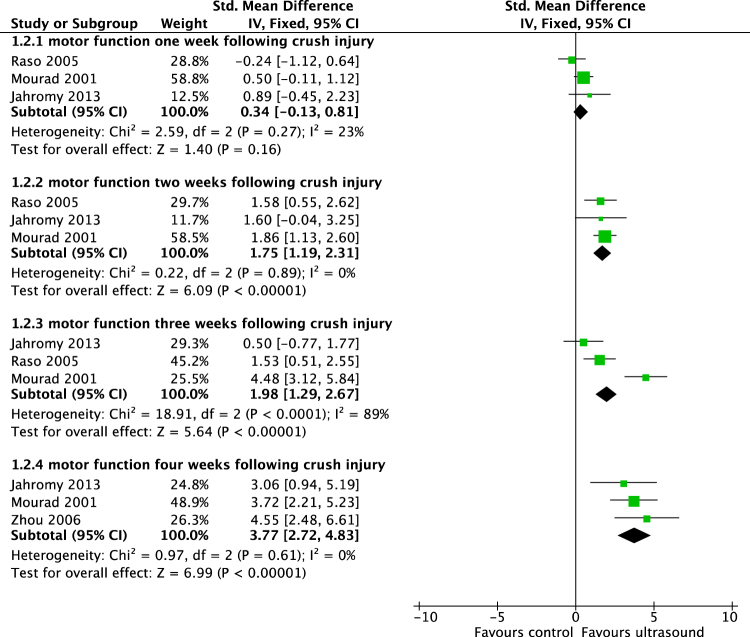
Meta-analysis. Standardized mean differences of walking track performance of 200–500 mW/cm^2^ US treated compared to sham or untreated animals at specific time points following crush injury.

**Figure 4:**
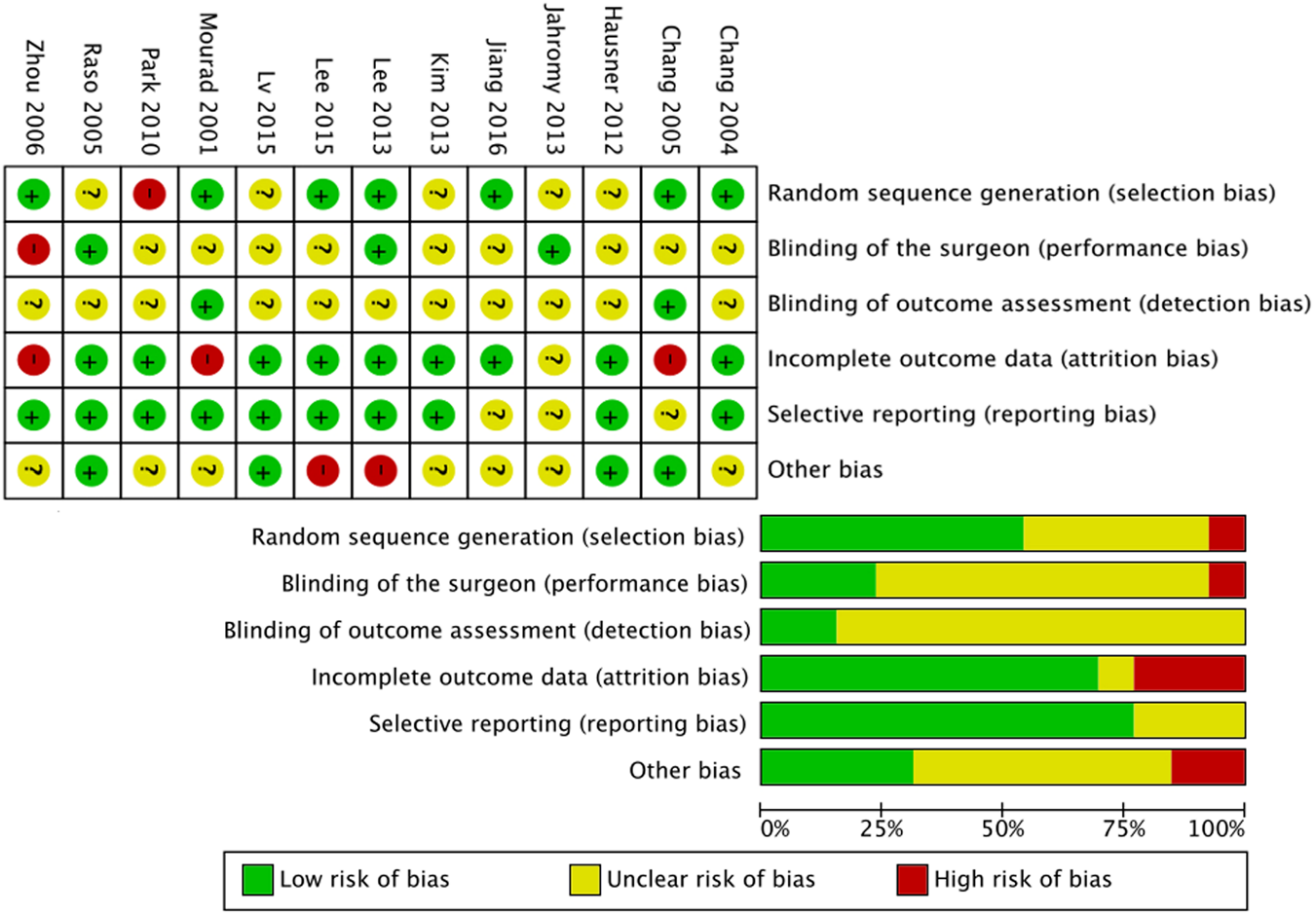
Risk of bias assessment of all included studies. The PRISMA guidelines require an analysis of potential biases, which would lead to under- or overestimation of the true intervention effect. Referring to the PRISMA guidelines, the authors judged the risk of bias (low-, unclear-, high risk of bias) for the following items for each included study: Selection bias, blinding of the surgeon, detection bias, attrition bias, reporting bias and other bias. Shown are the authors’ judgments about each risk of bias item for each study (upper part) and as percentages across all included studies (lower part).

To facilitate the interpretation of the effect estimates, we calculated the differences in means from the standardized mean differences that are shown in Figs 2 and 3, as described in the statistical analysis and data synthesis section. The methodological quality of each included study is shown in Fig. 4.

**Figure 5:**
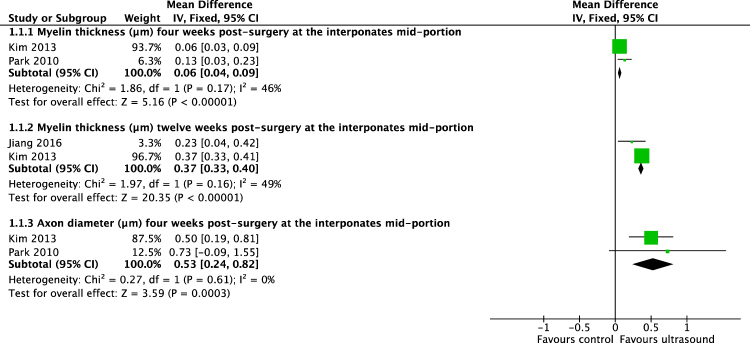
Meta-analysis. Differences in means of multiple histomorphometrical parameters of 400–500 mW/cm^2^ US-treated animals compared to sham or untreated animals on various time points following nerve reconstruction via nerve conduit.

Meta-analysis of included studies demonstrated that improved the motor function, as measured by the sciatic functional index (SFI)-scale, in axotomized rats two weeks after crush injury (difference in means [DM], 95% confidence interval[CI], fixed effect: 19,03 [13,2 to 25,6], I^2^ = 0%, 71 rats) and four weeks after crush injury (DM[95%CI], fixed effect: 7,4 [5,4 to 9,5], I^2^ = 0%, 47 animals) (Fig. 3). Following conduit or autograft implantation, US improved motor function on the SFI-scale as early as four weeks after surgery (DM[95%CI], fixed effect: 6,1 [4,6 to 7,7], I^2^ = 0%, 26 rats)(Fig. 2). Additionally, USincreased the diameter and myelin thickness of myelinated nerve fibers inside biodegradable nerve conduits, four weeks after implantation (diameter: DM[95%CI], fixed effect: 0,53 µm [0,42 to 0,82 µm], I^2^ = 0%, 12 animals; myelin thickness: DM[95%CI], fixed effect: 0,06 µm [0,04 to 0,09 µm], I^2^ = 46%, 12 animals) (Fig. 5). Likewise, three months following nerve repair by implantation of a custom made polylactic acid conduit, nerve conduction velocity was accelerated by US (DM[95%CI], fixed effect: 2,82 m/s [1,37 to 4,28 m/s], I^2^ = 0%, 16 animals).

**Figure 6:**
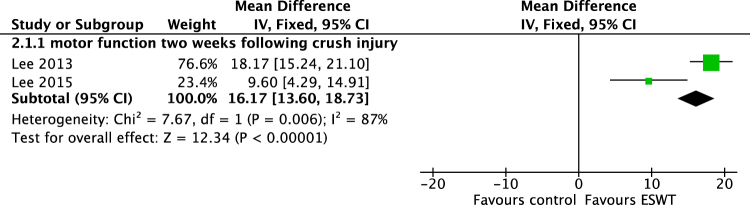
Meta-analysis. Difference in means of walking track performance of ESW-treated animals compared to untreated animals two weeks following crush injury with a considerable heterogeneity of I^2^ = 87% (n = 50).

ESWT showed to improve motor function on SFI-scale in axotomized rats two weeks after injury (DM[95%CI], fixed effect: 16,17 [13,6 to 18,73], 50 animals) but with considerable heterogeneity of I^2^ = 87% (Fig. 6). Following a single treatment, the improvements were only evident temporarily and not evident three months postoperatively.

## Discussion

Despite optimal surgical treatment, the functional outcomes following peripheral nerve injury are often unsatisfactory, necessitating additional treatment strategies. External stimulation following surgery could provide easily applicable therapies to accelerate axonal regeneration and thereby induce faster and improved recovery.

In preclinical animal studies, external stimulation such as US and ESWT have shown their potential to improve the regeneration of bones, tendons, intervertebral discs, ligaments and articular cartilage^13–16,36,37^. In humans, the positive effects of market-approved devices have been proven for conditions such as acute fractures^12^, plantar fasciitis^38^ and skin re-epithelization^39^. However, these modalities have not been explored clinically for peripheral nerve injuries yet. This study aimed to provide the first systematic review and meta-analysis of preclinical animal studies on the *in-vivo* effects of US or ESWT on axonal regeneration following peripheral nerve injury.

Ultrasound is defined as sound waves with frequencies above human’s auditory threshold. Preclinical and clinical investigations have shown that lower ultrasound intensities stimulate tissue regeneration by transmission of mechanical energy^12–14^. These positive effects are postulated to result from micro scale turbulences of inter- and intra-cellular fluids near vibrating structures, termed the acoustic streaming effect, and affect cellular membrane permeability and diffusion rates of transmembrane channels^40^. In recent animal studies, examining the nerve regeneration on a molecularbiological level, US was found to improve the early inflammatory response, accelerate Wallerian degeneration, enhance expression of essential growth factors like nerve growth factor (NGF) and ciliary neurotrophic factor (CNTF) and increase the number and activity of supporting Schwann cells in the absence of negative side effects^23,26,41–44^. To analyze the *in-vivo* effect of US in injured peripheral nerves, we reviewed the available literature and included ten preclinical animal studies including a total of 445 rats. Our findings indicate that ultrasound improved functional regeneration following sciatic nerve injury. These improvements occurred early after injury and were maintained throughout the observation period of two months in axonotmetic and three months in autografted animals. Electromyographic analyses suggest that this may result from accelerated axonal regeneration and earlier reinnervation of the target muscles. This is in accordance with previous findings of higher muscle weight, increased muscle fibers cross-sectional area and less muscle-fibrosis in US-treated animals^24,32^. Histomorphological analyses in US-treated animals found higher nerve fiber density, larger axons and thicker myelin sheaths distal to the crush lesion site and inside implanted conduits and autografts. Accordingly, US-treated rats had significantly faster nerve conduction velocities. Both, the morphological and the electrophysiological improvements occurred early and were maintained throughout the six-month observation period. These findings correspond with the enhanced expression of NGF and CNTF found in ultrasound treated nerves^26,41^. NGF is known to promote axonal sprouting whereas CNTF prevents motor neuron degeneration after axotomy and accelerates the early phase of axonal regeneration^45–50^. Similar effects have been found in retrograde labeling studies, as a higher number of axons successfully regenerated through a nerve conduit following US treatment^32^.

Previous preclinical studies varied widely in applied ultrasound parameters such as frequency, intensity, duration and treatment interval. Beneficial effects have been described even for comparatively strong doses as daily US application of even five minutes (500 mW/cm^2^) in the absence of negative effects^24^. To identify the most efficacious therapeutic regimen, we used subgroup analysis and found intensities of 200–300 mW/cm^2^ and 400–500 mW/cm^2^ to positively affect all investigated aspects of nerve regeneration. In direct comparison 200–300 mW/cm^2^ intensity was superior in motor function, axon number and compound muscle action potential^24,26,27^. Overall, the included US-studies have a minor risk of bias (Fig. 4). Furthermore, they provide significant evidence for US to accelerate axonal regrowth, to increase the number of axonal projections, to support early reinnervation of denervated muscle and to improve nerve conduction velocity after axonotmetic as well as after neurotmetic nerve injury.

ESWT is similar to US but uses different characteristics as single, predominantly positive pressure waves with high amplitudes, short duration and rapid rise times. Its positive effects have been described for orthopedic disorders, myocardial ischemia, and erectile dysfunction^21,51,52^. The underlying mechanisms have been identified as enhanced angiogenesis, enhanced growth factor synthesis and modulation of the inflammatory response^53–55^. For nerve regeneration, *in-vitro* shock wave therapy was reported to increase the proliferation rate and expression of regenerative phenotype-associated markers like glial fibrillary acidic protein and c-Jun in Schwann-cells^56^. However, likewise to US the *in-vivo* effects on nerve regeneration are only partly investigated and understood. Our results suggest that ESWT may improve early nerve regeneration but with no significant improvements remaining after twelve weeks. For example, Hausner *et al*. reported significantly superior motor function and higher nerve fiber numbers in single-ESW-treated subjects in the early phase of regeneration, that were, however, not statistically significant after three months compared to control. These temporary improvements may potentially result from accelerated axonal regrowth and thus earlier muscular reinnervation rather than an enhanced absolute number of sprouts crossing the nerve gap^29^. This agrees with the findings of faster nerve conduction in the ESW-treated animals compared to control, three months after surgery, since early muscular reinnervation stimulates maturing of the newly grown axons and consecutively their myelination. All reviewed ESWT-studies used approximately identical parameters (Table 2). However, the interval and the total number of treatments varied between one single-treatment immediately after surgery to five treatments per week for two weeks. In conclusion, because of the merely temporary effect and a high bias rating in two of three included studies, there is currently no evidence that ESWT can promote peripheral nerve regeneration.

**Table 2:** Experimental settings of the included studies. Listed are the key elements of all analyzed studies including initial nerve injury and type of surgical intervention, applied therapy, therapeutic regimen, animal number, observation period and reported predefined outcome.

Study	Nerve defect	Therapy	Therapeutic regimen	Included Animals (treated + untreated)	Observation period	Outcomes
Chang^30^	Conduit (12 mm)	US	pulsed_,_ (20%DC), 200 mW/cm^2^, 1 MHz, 5 min, 12×/2weeks	N = 48	6 weeks	Number and mean area of myelinated axons inside the conduit in week 6
Chang^31^	Conduit (17 mm)	US	pulsed (20%DC), 300 mW/cm^2^, 1 MHz, 5 min, 12×/2weeks	N = 48	8 weeks	Number and mean area of myelinated axons inside the conduit in week 8
Jahromy^26^	Sciatic nerve crush	US	continuous, 200 mW/cm^2^, 3, 3 MHz, 2 min, 12×/4weeks	N = 70	4 weeks	MF 12×/30d; CMAP in week 1, 2, 3 and 4
Jiang^24^	Reverse sciatic autograft (10 mm)	US	pulsed (20%DC), 250 or 500 mW/cm^2^, 1 MHz, 5 min, 7×/week until sacrifice	N = 60	12 weeks	MF in week 2, 4, 6, 8 and 12; CMAP in week 12; number and myelin thickness of myelinated axons inside the autograft in week12
Kim^32^	Conduit (12 mm)	US	pulsed (20%DC), 400 mW/cm^2^, 1 MHz, 2 min, 1×/week until sacrifice	N = 36	24 weeks	Myelin thickness and diameter of myelinated axons in week 4, 12 and 24, NCV in week 12 and 24
Lv^22^	Conduit (12 mm)	US	pulsed (20%DC), 300 mW/cm^2^, 1 MHz, 5 min, 14×/2weeks	N = 16	12 weeks	MF in weeks 4 and 12; NCV in week 12
Mourad^27^	Sciatic nerve crush	US	continuous, 500 mW/cm^2^ + 1 MHz, 250 mW/cm^2^ + 1 MHz, 250 mW/cm^2^ + 0,25 MHz, 1 min, 12×/4weeks	N = 53	4 weeks	MF on day 7, 14, 16, 18, 21, 24, 26, 28 and 30
Park^33^	Conduit (12 mm)	US	pulsed (20%DC), 400 mW/cm^2^, 1 MHz, 2 min, 8×/8weeks	N = 30	3 weeks	Myelin thickness and axon diameter in week 4 and 8
Raso^23^	Sciatic nerve crush	US	pulsed (20%DC_3_), 400 mW/cm^2^, 1 MHz, 2 min, 10×/10days	N = 20	3 weeks	MF in week 1,2, and 3; nerve fiber density in week 3
Zhou^25^	Sciatic nerve crush	US	continuous, 250 mW/cm^2^, 1 MHz, 1 min, 7×/week until sacrifice	N = 64	8 weeks	MF and NCV in week 4,6 and 8; density of myelinated axons in week 2, 4, 6 and 8
Hausner^29^	Reverse sciatic autograft (8 mm)	ESWT	300 impulses, 3 Hz, 0.1 mJ/mm^2^, once after surgery	N = 40	12 weeks	Number of myelinated axons and NCV in week 3 and 12; MF in week 4, 6, 8, 10 and 12
Lee^34^	Sciatic nerve crush	ESWT	300 impulses, 3 Hz, 0.09 mJ/mm^2^, 6×/2weeks	N = 40	2 weeks	MF on day 1 and 14
Lee^35^	Sciatic nerve crush	ESWT	300 impulses, 3 Hz, 0.09 mJ/mm^2^, 10×/2weeks	N = 30	6 weeks	MF on day 1, 7, 14, 21, 28, 35 and 42

US, ultrasound therapy; ESWT, extracorporeal shock wave therapy; DC, duty cycle; MF, voluntary motor function; CMAP, compound muscle action potential; NCV, nerve conduction velocity.

Limitations of this meta-analysis are the number of available studies and the included studies’ risk of bias. The analyzed studies did not investigate US or ESWT following complete nerve transection and primary repair, which would represent the most frequent clinical situation. Moreover, following implantation of a conduit or autograft, the mid-portion of the respective interponate was analyzed for the histomorphological evaluation, whereas from a clinical perspective, the histomorphology distal to the interponate is more relevant. Since all analyzed preclinical studies were conducted in small experimental animals, the optimal application area in bigger subjects remains unclear. However, in regard to the underlying mechanisms, the optimal clinical application is presumably located above the regenerating axons, which can be clinically localized using the Hoffmann-Tinel sign.

Nevertheless, the promising findings of US therapy on nerve regeneration imply a wide range of application possibilities in peripheral nerve surgery. Especially proximal, high-level injuries with a long recovery time might benefit from US following nerve surgery due to faster axonal regeneration, reduced denervation time and therefore faster recovery. Potential clinical investigations could significantly profit from the experience of market-approved devices used in other disciplines. These transducers have been shown to be cost-effective and are thus recommended by the Medical Technology Guidance of UK National Institute for Health and Care Excellence (NICE) as they offer benefits to patients at lower costs compared with current practice in the treatment of long bone fractures with non-union^57^. The NICE Assessment Centre has found no clinical studies that reported any device-related adverse events and identified no significant safety concerns about the recommended US-transducers^57^. The devices can usually be self-applied and have excellent compliance rates of around 90%^58^. Moreover, the integrated software of FDA approved transducers allows easy clinical supervision of outpatient treatment.

## Conclusion

Overall, there is significant evidence for US to experimentally promote regeneration after axonotmetic and neurotmetic nerve injury. On the contrary, the available ESWT studies have several limitations and can therefore not provide conclusive evidence for ESWT to promote nerve regeneration. Given the preclinical benefits in the absence of any negative side effects, low-intensity ultrasound devices, as approved by the FDA, should be investigated clinically in humans as an adjunct therapy following nerve surgery.

## Methods

A systematic review and meta-analysis of experimental ultrasound and shock wave stimulation in peripheral nerve surgery was conducted according to the PRISMA statement^59^.

### Search strategy and eligibility criteria

As recommended by the PRISMA guidelines, the authors designed a systematic search strategy in September 2016 for the databases PubMed (see supplemental material), Web of Science core collection and BIOSIS, to identify all potentially relevant studies for this review. The date of the last search for each database was October 3^rd^, 2016. The results of this systematic search were screened for possible inclusion against a predetermined checklist of inclusion criteria (Table 1). All experimental animal studies in rats comparing ultrasound therapy or extracorporeal shock wave treatment with sham or no intervention groups after peripheral motor nerve lesions were possibly eligible for inclusion into the analysis. Based on previous reports investigating the efficacy of ultrasound, we focused on studies using a maximum intensity of 500 mW/cm^2^, since higher intensities did not beneficially affect recovery following nerve injury in previous studies^24,27^. Studies not meeting the inclusion criteria were excluded and are listed in Table 3. Additional information about the detailed search strategies is located in the Supplemental Material.

**Table 3:** Excluded studies. List of all studies excluded from the analyses following full-text assessment, and the underlying detailed exclusion criteria.

Study	Reasons for exclusion
Chen^41^	Similar data set to previous publication
Crisci^42^	Incomplete data reporting and inappropriate outcome assessment
Hong^65^	Non-randomized allocation of heterogenic animals, non-standard injury models, high dropout rate and ultrasound application without coupling medium
Mense^66^	Reported none of the predefined outcomes of interest
Oliveira^67^	Incomplete data reporting, non-standardized nerve injury model, non-standardized measurement procedures and duration of experiments

### Study selection and data extraction

First, titles and abstracts were screened to identify all potentially eligible studies. Studies meeting the inclusion criteria were obtained in full text and assessed thoroughly for eligibility. The reference lists of the included literature were used to identify further relevant publications. For data extraction and analysis, the voluntary motor function was chosen as the primary outcome parameter. Therefore, we used the rats’ walking track performance as a frequently applied tool to assess motor function following nerve injury in the rat sciatic nerve-model. Here the hind paw foot prints of the injured leg are recorded and measured. De Medinaceli formulated the sciatic functional index (SFI)-scale to quantify the walking track performance after sciatic injury in rat^60^. This well-established SFI-scale ranges from values around zero in non-injured animals to a low of around −100 immediately after complete loss of axonal continuity^61^. Secondary outcomes were neuro-histomorphological parameters as diameter of myelinated axons, myelin sheath thickness and density or number of myelinated nerve fibers distal to the lesion site or inside the implanted interponate as well as neurophysiological parameters such as NCV or CMAP of target muscles. All relevant data including the time period between nerve injury and outcome analyses were extracted (Table 4) and the included studies were evaluated for methodological quality, guided by the Cochrane Handbook for Systematic Reviews of Interventions^62^ (Fig. 4). The risk of bias was assessed for the following aspects: random sequence generation (selection bias), blinding of the surgeon (performance bias), blinding of outcome assessment (detection bias), incomplete outcome data (attrition bias), selective reporting (reporting bias) and other bias like non-standardized nerve lesion models.

**Table 4:** Extracted data. Following the PRISMA guidelines, all included studies were systematically analyzed to extract the following experimental aspects. These data provided a basis for the subsequent subgroup analyses and ensured the inter-study comparability.

Extracted data
• Number, sex, age and weight of included animals
• Type of nerve injury and surgical intervention
• US treatment regimen parameters: intensity, frequency, mode of emission (continuous or pulsed), duty cycle, duration and interval of treatment
• ESWT sonication regimen as number of applied impulses, energy, frequency and interval of treatment
• Reported outcomes of interest and duration of experiment

### Statistical analysis and data synthesis

The meta-analysis was performed using Cochrane statistical software Review Manager 5.3 (Cochrane Collaboration, Copenhagen, the Nordic Cochrane Centre). US-studies were clustered in two application intensities, ranging from 200–300 mW/cm^2^ and 400–500 mW/cm^2^ to enable dose-dependent comparison of the various studies, since previous reports detected significant differences in several outcomes of interest between groups treated with these intensities^24^. Thereafter, subgroup analyses were conducted according to the intensity of ultrasound treatment. The inverse variance method in a fixed effect analysis-model was applied and the results were expressed as difference in means (DM) with 95% confindence interval (CI) for continuous outcomes. When different scales were used to measure the same outcome, we calculated the standardized mean difference (SMD) and calculated the DM afterwards on a suitable scale by multiplying the SMD by a typical among-person standard deviation for the target scale, as recommended by the Cochrane Handbook for Systematic Reviews^63^. When standard error of means (SE) was reported instead of standard deviation (SD) we calculated the SD by multiplying the SE by the square root of the sample size:

$$SD=SE\cdot \sqrt{n}.$$

To calculate the average intervention effect across included studies, we weighted the estimated intervention effects of the individual studies according to the width of their confidence intervals^64^. Thereby the studies with higher precision (narrower confidence intervals) have more influence on the average intervention effect.

If relevant data was not reported or incomplete in a study, the risk of bias was consecutively rated higher. We assessed experimental heterogeneity across included studies using the Chi^2^ and the I^2^ test^64^. Additionally, we reviewed the outcome data individually and summarized it in a narrative form in “effects of interventions” section. A significance level of 5% was used (p < 0,05).

### Data availability

The datasets analyzed during this systematic review and meta-analysis are available from the corresponding author on reasonable request.

## Electronic supplementary material

Supplemental file
