# Supplementary material for: Ultrasound and shock-wave stimulation to promote axonal regeneration following nerve surgery: a systematic review and meta-analysis of preclinical studies
Source: Sci Rep. 2018 Feb 16;8:3168. doi: 10.1038/s41598-018-21540-5 (PMC5816639; doi:10.1038/s41598-018-21540-5)
Supplement: Supplementary file 1 — Supplemental file [file 41598_2018_21540_MOESM1_ESM.pdf]

Ultrasound and shock-wave stimulation to promote axonal regeneration following nerve surgery: a systematic review and meta-analysis of preclinical studies

Simeon C. Daeschler[1], Leila Harhaus MD[1], Philipp Schoenle MD[1], Arne Böcker MD[1], Ulrich Kneser MD[1], Konstantin D. Bergmeister MD PhD[1] *

[1] Department of Hand, Plastic and Reconstructive Surgery, Burn Center, Department of Plastic and Hand Surgery, University of Heidelberg, BG Trauma Hospital Ludwigshafen, Ludwigshafen, Germany

Supplemental Material

Excluded studies

We excluded a total of six studies after review of the full-text articles due to non-standardized injury models or insufficient data reporting. The detailed exclusion criteria are listed in the supplementary table one.

Search strategy

Database: PubMed, date of the last search: 3rd October 2016

1. "peripheral nerves"[MeSH Terms]
2. nerve recover*
3. "nerve repair"
4. "nerve crush"
5. nerve lesion*
6. "nerve regeneration"
7. nerve injur*
8. axotom*
9. nerve degeneration[MeSH Terms]
10. nerve regeneration[MeSH Terms]
11. nerve crush[MeSH Terms]

12. "axotomy"[MeSH Terms]
13. peripheral nerve injury[MeSH Terms]
14. "Mononeuropathies"[Mesh]
15. #1 - #14 OR
16. ultrasound therap*
17. ultrasound stimul*
18. therapeutic ultrasonograph*
19. therapeutic ultrasound*
20. low intensity ultrasound*
21. pulsed ultrasound*
22. eswt
23. shockwave*
24. shock wave*
25. lius
26. lipus
27. "high energy shock waves/therapeutic use"[MeSH Terms]
28. "ultrasonography/therapeutic use"[MeSH Terms]
29. "ultrasonography/therapy"[MeSH Terms]
30. "ultrasonic waves/therapeutic use"[MeSH Terms]
31. "ultrasonics/therapy"[MeSH Terms]
32. "ultrasonics/therapeutic use"[MeSH Terms]
33. ultrasonic therapy[MeSH Terms]
34. #16 - #33 OR
35. #15 AND #34
36. Animals[Mesh:noexp]
37. #35 AND #36

Supplementary Tables

Study	Reasons for exclusion
**Chen 2010**[41]	Similar data set to previous publication
**Crisci 2002**[42]	Incomplete data reporting and inappropriate outcome assessment
**Hong 1988**[65]	Non-randomized allocation of heterogenic animals, non-standard injury models, high dropout rate and ultrasound application without coupling medium
**Mense 2013**[66]	Reported none of the predefined outcomes of interest
**Oliveira 2012**[67]	Incomplete data reporting, non-standardized nerve injury model, non-standardized measurement procedures and duration of experiments

Supplementary Table 1. Excluded studies. List of all studies excluded from the analyses following full-text assessment, and the underlying detailed exclusion criteria.
